# The Multicopper Ferroxidase Hephaestin Enhances Intestinal Iron Absorption in Mice

**DOI:** 10.1371/journal.pone.0098792

**Published:** 2014-06-04

**Authors:** Brie K. Fuqua, Yan Lu, Deepak Darshan, David M. Frazer, Sarah J. Wilkins, Natalie Wolkow, Austin G. Bell, JoAnn Hsu, Catherine C. Yu, Huijun Chen, Joshua L. Dunaief, Gregory J. Anderson, Chris D. Vulpe

**Affiliations:** 1 Department of Nutritional Science and Toxicology, University of California, Berkeley, Berkeley, California, United States of America; 2 Iron Metabolism Laboratory, QIMR Berghofer Medical Research Institute, Brisbane, Queensland, Australia; 3 FM Kirby Center for Molecular Ophthalmology, Scheie Eye Institute, University of Pennsylvania, Philadelphia, Pennsylvania, United States of America; 4 Medical School, Nanjing University, Nanjing, Jiangsu Province, China; 5 School of Chemistry and Molecular Bioscience, University of Queensland, Brisbane, Queensland, Australia; CINVESTAV-IPN, Mexico

## Abstract

Hephaestin is a vertebrate multicopper ferroxidase important for the transfer of dietary iron from intestinal cells to the blood. Hephaestin is mutated in the sex-linked anemia mouse, resulting in iron deficiency. However, sex-linked anemia mice still retain some hephaestin ferroxidase activity. They survive, breed, and their anemia improves with age. To gain a better understanding of the role of hephaestin in iron homeostasis, we used the Cre-lox system to generate knockout mouse models with whole body or intestine-specific (Villin promoter) ablation of hephaestin. Both types of mice were viable, indicating that hephaestin is not essential and that other mechanisms, multicopper ferroxidase-dependent or not, must compensate for hephaestin deficiency. The knockout strains, however, both developed a microcytic, hypochromic anemia, suggesting severe iron deficiency and confirming that hephaestin plays an important role in body iron acquisition. Consistent with this, the knockout mice accumulated iron in duodenal enterocytes and had reduced intestinal iron absorption. In addition, the similarities of the phenotypes of the whole body and intestine-specific hephaestin knockout mice clarify the important role of hephaestin specifically in intestinal enterocytes in maintaining whole body iron homeostasis. These mouse models will serve as valuable tools to study the role of hephaestin and associated proteins in iron transport in the small intestine and other tissues.

## Introduction

Every day, billions of iron atoms from the diet must be transferred from intestinal cells to the blood in order to maintain iron balance. The transfer of iron across biological membranes is usually associated with the oxidation or reduction of the iron, and current evidence supports an important role for the vertebrate multicopper ferroxidase (MCF) hephaestin (HEPH) in the export of iron from intestinal enterocytes [1]. HEPH is hypothesized to oxidize ferrous iron from the only known intestinal iron exporter, ferroportin (FPN1), a multi-pass membrane protein that has been demonstrated in other cell types to require a ferroxidase to function [2]–[4]. This catalyzed oxidation step also ensures that adequate iron is available to bind to its carrier in the blood, transferrin, which, under physiological conditions, only binds ferric iron [5], [6]. HEPH is the only known MCF expressed in intestinal enterocytes [7]. While ceruloplasmin (CP), a MCF paralog of HEPH found in both circulating and glypiated (GPI-linked) forms, has been shown to play an analogous role in iron oxidation and release from other cell types, humans and mice that lack CP do not appear to have defects in iron absorption except, as has been shown in mice, in situations of severe iron need [8]. HEPH is highly expressed along the length of the intestinal tract, and lower levels of expression have been reported in a variety of tissues including the central nervous system, lungs, heart, and exocrine pancreas [1], [9]–[13]. Unlike CP, no HEPH expression has been detected in the liver or serum [1], [9].

HEPH was identified in 1999 by Vulpe et al. who mapped the mutated gene responsible for the phenotype of the sex-linked anemia (*sla*) mouse [1]. The *sla* mouse model arose in the 1950s in an irradiated mouse colony and has a deletion in the gene encoding HEPH (*Heph*), corresponding to a large internal portion of the protein [14]. *Sla* mice have a defect in iron absorption [15], [16]. While dietary iron uptake into intestinal enterocytes appears to be normal, iron is not released properly into the body, resulting in iron loading in the duodenum and a moderate to severe hypochromic, microcytic anemia. The anemia generally improves with age, as might be expected since iron needs are greatest during the rapid growth that accompanies early life [17]. Nevertheless, *sla* mice still remain iron-deficient throughout life relative to wild-type controls [18].

The phenotype of *sla* mice indicates that HEPH plays an important role in iron absorption. However, *sla* mice still produce a mutant HEPH protein at levels approximately 25% that of wild-type that, remarkably, retains significant ferroxidase activity [19]. It thus remained unclear if the *sla* mouse represented a complete null or a hypomorph. If HEPH is not absolutely required, then either one or more other ferroxidases must be involved, or there must be another mechanism for iron absorption that does not involve a catalyzed oxidation step. To determine whether or not HEPH is absolutely required for intestinal iron absorption, we generated both whole body and intestine-specific HEPH knockout mice (Heph^-/-^ and Heph^int/int^ mice, respectively). The Heph^int/int^ model was designed to enable the study of the specific role of HEPH in the intestine, since HEPH is expressed at low levels in other tissues as well. We also directly compared the phenotypes of Heph^-/-^ and *sla* mice in order to determine potential differences in these models which could provide insights into HEPH function.

## Materials and Methods

### Ethics statement

All work performed was in accordance with the National Institutes of Health (NIH) guidelines, as described in the Guide for the Care and Use of Laboratory Animals of the NIH, and with approval from the Office of Laboratory Animal Care at the University of California, Berkeley, and the QIMR Berghofer Medical Research Institute Animal Ethics Committee. *In vivo* experiments are reported here in accordance with the ARRIVE guidelines [20]. All efforts were made to minimize animal suffering.

### Mouse models

We used the Cre-lox system to create the Heph^-/-^ and Heph^int/int^ mouse models [21]. It is important to note that *Heph* is located on the X chromosome, and thus males only carry one allele of *Heph*. The generation of the *Heph* floxed mouse strain (C57BL/6-Heph^tm1.1Jdun^, referred to here as Heph^fl/fl^ in general and Heph^fl/y^ for males) has been recently described [22]. In these mice, LoxP sites flank exon 4 of the *Heph* gene. We crossed Heph^fl/fl^ mice (kindly provided by Professor Joshua Dunaief at the University of Pennsylvania and previously backcrossed onto the C57BL/6J background) with C57BL/6J transgenic mice bearing the Cre recombinase transgene driven by either the EIIa promoter, which is ubiquitously active (“EIIa-Cre” mice, B6.FVB-Tg(EIIa-Cre)C5379Lmgd, The Jackson Laboratory), or the Villin promoter, which is only activated in the intestine (“Villin-Cre” mice, B6SJL-Tg(Vil-Cre)997Gum, The Jackson Laboratory), in order to generate the Heph^-/-^ and Heph^int/int^ knockout strains, respectively. Expression of Cre recombinase leads to excision of the region in the DNA between the LoxP sites (exon 4 of *Heph*). Exon 4 is the second protein coding exon in *Heph* and contains residues required for the trinuclear copper cluster site that is needed for ferroxidase activity [22], [23]. Removal of this exon also causes the remaining protein-coding sequence to be out of frame and introduces an early stop codon. After germline knockout was achieved in the Heph^-/-^ strain, the EIIa-Cre recombinase transgene was bred out of the line.

To generate male Heph^-/y^ and wild-type (WT) littermates for experiments, heterozygous Heph^+/-^ females were bred with Heph^-/y^ males. Male Heph^int/y^ and Heph^fl/y^ littermate controls were generated by crossing Heph^fl/fl^ females with Heph^int/y^ males. In this latter cross, all mice and progeny were homozygous or hemizygous for floxed *Heph* alleles, and the Heph^int/y^ breeders and male Heph^int/y^ and female Heph^int/int^ progeny carried only one copy of the Villin-Cre transgene.

The EIIa- and Villin-Cre mouse strains were obtained from The Jackson Laboratory (Bar Harbor, ME). The *sla* mice were from stocks maintained at QIMR Berghofer but were originally from The Jackson Laboratory (B6.Cg-Heph*^sla^*/J, stock # 001180). Tamoxifen-inducible FPN1 intestinal knockout mice (Fpn1^int/int^) were generated as previously described by crossing Fpn1^fl/fl^ mice on a mixed background (kindly provided by Professor Nancy Andrews at Duke University) with mice bearing the VilERT2-Cre recombinase transgene (Tg(Vil-Cre/ERT2)23Syr) [24]. The VilERT2-Cre mice were originally created by Dr. Sylvie Robine at the Institut Curie in France and were kindly provided to us by Professor Nicholas Davidson at Washington University. To induce intestinal *Fpn1* knockout in *Fpn1* floxed mice bearing the VilERT2-Cre transgene, pups (7 days old) were injected once daily for three days with tamoxifen. The tamoxifen solution was prepared fresh on the first day of injection by dissolving tamoxifen powder (Sigma, St. Louis, MO) in 100% ethanol to 50 mg/mL. The dissolved tamoxifen was then diluted to 8.3 mg/mL in sunflower seed oil (Sigma) and then injected subcutaneously at 75 µg tamoxifen/g body weight. The remaining tamoxifen solution was stored at 4°C, and then warmed by hand and vortexed to ensure solubilization prior to use on subsequent days. Mice were closely monitored for any adverse reactions. Mice for all studies were on the C57BL/6J background unless otherwise noted.

### Animal husbandry

Mice were maintained on a standard rodent chow diet containing approximately 160 ppm iron (Norco Stockfeeds, South Lismore, NSW, Australia) or, for iron absorption studies as indicated, an iron-deficient diet made in-house and described previously [9]. Mice fed the iron-deficient diet and dosed with ^59^Fe were provided with iron-free deionized water and housed in grid-bottom cages designed to minimize coprophagia. Unless otherwise noted, all mice were allowed unlimited access to food and water.

### Tissue collection

Mice were euthanized by CO_2_ gas inhalation or anesthetized by a single intraperitoneal (IP) injection of 44 mg/kg ketamine and 8 mg/kg xylazine in saline. Blood was then collected from the posterior vena cava and heart. Approximately 250 µL of blood was collected into potassium EDTA tubes (Terumo Corporation, Tokyo, Japan, cat #3T-MQK) for whole blood analysis. Tissues were harvested, trimmed to remove connective tissue, and either immediately snap frozen in liquid nitrogen for future RNA/protein analyses or incubated in 10% phosphate buffered formalin overnight at 25°C for future processing for Perls' Prussian blue staining for iron. Intestinal enterocytes were collected as previously described [25]. In brief, the duodenal segment was trimmed of connecting tissue and cut open longitudinally. The segment was then washed in ice-cold phosphate buffered saline (PBS), pH 7.4, followed by incubation with agitation in ice-cold PBS containing 1.5 mM EDTA to chelate calcium and disrupt tight junctions which allows the enterocytes to come free from the underlying tissues. The cells were then pelleted by centrifugation at 500×*g* for 5 minutes at 4°C, and the supernatant was discarded. Blood and other non-enterocyte cells, if present, form a small visible layer on top of the enterocytes after centrifugation. This layer was removed by aspiration if present. Enterocytes were then resuspended in cold PBS by inversion and centrifuged again. The wash and centrifugation steps were repeated twice more, and the cells were then snap frozen. Previous analyses have indicated that >95% of the cells collected by this method are enterocytes [25].

### PCR genotyping

Mice were routinely genotyped by PCR using DNA obtained from a 1–2 mm piece of the distal tail tip or from a small ear punch sample. For genotyping of other tissue samples, a small piece (∼2–3 mm^3^) of frozen tissue was first homogenized in 500 µL ice-cold lysis buffer containing 25 mM tris(hydroxymethyl)aminomethane (Tris), pH 7.4, 125 mM NaCl, 0.1% sodium dodecyl sulfate (SDS), and 0.2% polysorbate 20 (Tween-20) by vigorous shaking with 1.4 mm ceramic beads (MO BIO Laboratories, Carlsbad, California, cat. #13113-325) in a Precellys24 tissue homogenizer (Bertin Technologies, France) at 4°C. DNA was prepared from tail tip and ear punch pieces or 5 µL of other tissue homogenates using the QuickExtract DNA Extraction Solution (Epicentre, cat #QE09050) as per the manufacturer's instructions, but with only 50 µL extract solution per sample. PCR was performed using MangoTaq DNA Polymerase master mix (Bioline, cat #BIO-21083) as per the manufacturer's instructions. Primers, annealing temperatures, and expected product sizes are described in Table 1.

**Table 1:** Genotyping primers and expected product sizes.

Target	Primer	Primer sequence (5′ to 3′)	Annealing temperature (°C)	Expected products
***Heph*** ** WT/floxed**	Primer-F Primer-R	GACCTAGGAAGGAGAATCATGAG TTTGCGAGCCGACCTTACACC	55	240 bp WT allele; 300 bp floxed allele
***Heph*** ** knockout**	Primer-F Primer-R	TGTCATTCAATTCCTGGAAAA CCAAGAAAATGGAGAAAGAGG	55	230 bp
***Sla*** ** (absent)**	Primer-F Primer-R	TGCATGTTCCTAAACCAGGA CAGCCTTGCACACCAGTAGA	55	370 bp (WT allele)
***Sla*** ** (present)**	Primer-F Primer-R	TGCATGTTCCTAAACCAGGA ATCTTGTTGCTCAGGCTGGT	55	480 bp
**EIIa-Cre**	Primer-F Primer-R	GCGGTCTGGCAGTAAAAACTATC GTGAAACAGCATTGCTGTCACTT	55	100 bp
**Internal control (55°C)**	Primer-F Primer-R	CTAGGCCACAGAATTGAAAGATCT GTAGGTGGAAATTCTAGCATCATCC	55	320 bp
**Villin-Cre**	Primer-F Primer-R	GTGTGGGACAGAGAACAAACC ACATCTTCAGGTTCTGCGGG	64	1100 bp
**Internal control (64°C)**	Primer-F Primer-R	CAAATGTTGCTTGTCTGGTG GTCAGTCGAGTGCACAGTTT	64	200 bp

### Protein and RNA analyses

A small frozen piece of each tissue was removed from low temperature storage and disrupted by vigorous shaking with 1.4 mm ceramic beads as described above. For RNA preparation, this homogenization step was performed in ice-cold TRIzol (Invitrogen, Melbourne, VIC, Australia) and RNA was extracted as per the manufacturer's instructions. Complementary DNA (cDNA) was synthesized using oligo(dT) primers and Moloney Murine Leukemia Virus reverse transcriptase (Invitrogen) as per the manufacturer's instructions. Real-time quantitative PCR (RT-qPCR) was performed using LightCycler (LC) 480 SYBR Green I Master Mix in an LC480 machine (Roche, Basel, Switzerland) as per the manufacturer's instructions, and the data were analyzed by calculating the concentration of each sample from its Ct value using a standard curve made from pooled sample cDNA as described previously [26]. The results were then normalized to the expression of the housekeeping gene hypoxanthine guanine phosphoribosyl transferase (*Hprt*). All primers (Table 2) were validated by melt-curve analysis, PCR product size analysis, and in most cases, by PCR product sequencing.

**Table 2:** RT-qPCR primers.

Target	Primer	Primer sequence (5′ to 3′)
***mHeph-1*** ** (unmodified region, exon 6**–**7 junction)**	Primer-F Primer-R	CATGCAATCAATGGGTTTGTCT TTTGCAACATCAGTGTGGTGTC
***mHeph-9*** ** (exon 4**–**5 junction)**	Primer-F Primer-R	TTGCGAGCCGACCTTACACC TGCCTCAGTGGGGGCATGACT
***mHamp1***	Primer-F Primer-R	AGAGCTGCAGCCTTTGCAC ACACTGGGAATTGTTACAGCATTTA
***mTfrc***	Primer-F Primer-R	GGTGTTGCGGCGAAGTCCAGT ACTCAGTGGCACCAACAGCTCC
***mHprt***	Primer-F Primer-R	GGACTGATTATGGACAGGA GAGGGCCACAATGTGATG
***mDmt1***	Primer-F Primer-R	TCATACCCATCCTCACGTTCAC GGTCAAATAAGCCACGCTAACC

For protein expression analysis by immunoblotting, tissue was homogenized in ice-cold lysis buffer (25 mM Tris-HCl, pH 7.2, 25 mM NaCl, 0.25% Tween-20, 0.25% NP-40, 0.1% SDS) with protease inhibitors as per the manufacturer's instructions (Complete protease inhibitor cocktail tablets, Roche, cat #11697498001). The homogenized sample was centrifuged at 16,000×*g* for 30 minutes at 4°C to pellet debris, and the supernatant was then collected and assayed for protein concentration using the Pierce BCA Protein Assay Kit (Thermo Fisher Scientific, Rockford, IL). Total protein (60–80 µg) was mixed with SDS loading buffer with fresh dithiothreitol (DTT) and heated at 75°C for ten minutes (with the exception of samples for FPN1 immunoblotting, which were not heated). The samples were then separated on 10% (for HEPH and FPN1) or 15% (for FTN) Tris-glycine SDS polyacrylamide denaturing gels, and then transferred to Immobilon-FL polyvinyl difluoride (PVDF) membranes (Merck, Kilsyth, Australia). Membranes were blocked for one hour at room temperature with shaking in blocking buffer (10% non-fat milk in Tris-buffered saline with 0.1% Tween- 20 (TBST)). Membranes were then incubated with the relevant primary antibody diluted in blocking buffer for one hour at room temperature (FTN [rabbit anti-human FTN, 1∶5000, cat #650771, ICN Biomedicals, Seven Hills, Australia]; HP [rabbit anti-HP C-terminus, 1∶1000, cat #HEPH11-A, Alpha Diagnostics, Owings Mills, MD]; HP [rabbit anti-HP D4 center, 1∶3000, produced in-house [27]]; Actin [mouse monoclonal anti-beta actin, 1∶10,000, cat #ab6276, Abcam, Cambridge, UK]; FPN1 [rabbit anti-FPN1, 1∶2500, cat #MTP11-A, Alpha Diagnostics]). The membranes were then washed with TBST and incubated for one hour at room temperature with secondary antibody (anti-rabbit HRP, 1∶8000, Merck, or anti-mouse HRP, 1∶10,000). The blots were washed in TBST and imaged on film or in a digital imager (LAS500, GE Healthcare) after incubation with the Western Lightning Plus-ECL Kit solutions (Perkin-Elmer, Glen Waverley, VIC, Australia). Densitometry was performed using the TotalLab Quant software (Newcastle upon Tyne, UK). For each sample, results for the protein of interest were normalized to that of actin. The results for each mouse group were then averaged and normalized to the average result for the control group.

### Tissue iron staining and quantitative tissue iron measurements

Tissue was paraffin processed, sectioned, and the sections stained for ferric iron using Perls' Prussian blue stain by the Histotechnology Facility at QIMR Berghofer [28]. For colorimetric non-heme iron quantification, a modification of the Torrance and Bothwell method was used [29]. Tissues were wrapped in aluminum foil and dried in an oven at 110°C. A small piece of the dried tissue (15–20 mg) was weighed in a 1.5 mL acid-resistant tube and 1 mL of an acid solution (3 M HCl, 0.6 M trichloroacetic acid) was added. A series of standards (100 µL final volume) was also made up containing 0–50 µg iron/mL acid solution. The samples and standards were then incubated for 20 hours in a 65°C water bath. Solutions were then vortexed, centrifuged briefly at maximum speed in a microcentrifuge, and allowed to cool. Freshly prepared chromogen reagent (one volume 0.1% bathophenanthroline disulfonic acid with 1% w/v thioglycolic acid, five volumes saturated sodium acetate, and five volumes iron-free water) was added to wells in a clear plastic 96-well plate (200 µL/well). Sample or standard (4 µL per well) was added and mixed by pipet. The plate was incubated at room temperature for 30 minutes and the absorbance at 535 nm was measured in a microtiter plate reader. The concentration of iron in the samples was then calculated based on the constructed standard curve.

### Blood analyses

Blood samples were analyzed on a Coulter Ac•T diff Analyzer using the Veterinary Application Software (Beckman-Coulter, Fullerton, CA) and reticulocytes were counted at the Pathology Department of the Royal Brisbane and Women's Hospital (Brisbane, Australia) using a Sysmex XE-5000 automated hematology analyzer (Roche Diagnostics, Castle Hill, NSW, Australia).

### Iron absorption assays

Whole animal absorption measurements were carried out by giving mice an oral dose of ^59^Fe followed by whole body counting. Mice initially maintained on a chow diet were switched to an iron-deficient diet and subsequently housed in cages with wire grid bottoms. After a specified period of time on the diet, mice were dosed by gavage with 100 µL of a freshly prepared solution containing 200 µM ferrous sulfate in 10 mM HCl, spiked with 3 µCi ^59^Fe radiolabeled iron (Perkin-Elmer, Waltham, MA, cat #NEZ037001MC) per dose. In the iron absorption study where mice were put on the iron deficient diet for one week prior to dosing, mice were fasted but provided with water *ad libitum* for approximately four hours both before and after dosing. The radiation in each mouse was measured using a RAM DA gamma counter with a PM-11 tube (Rotem Industries, Arava, Israel) at a fixed distance just after dosing, and then again five days later. The second reading was taken five days after dosing to give time for complete turnover of the intestinal epithelial cells, so that only ^59^Fe that had been transferred from enterocytes to the body would be detected [30]. After subtraction of the background counts, the percentage of the initial dose remaining in the mouse was calculated. Mice were then euthanized by ketamine/xylazine/saline IP injection as described under “Tissue collection”, and once unresponsive to paw pinch, the body cavity of the mouse was opened. The gastrointestinal (GI) tract was excised and placed in a 10 mL tube. The removed GI tract comprised the lower esophagus just proximal to the stomach down to the anus, and included connective tissue, pancreas and any contents inside the GI tract. The liver was then excised, wrapped in foil, and placed in an oven to dry as described above. Radiation in the GI tract was measured at the fixed distance from the gamma counter. After background subtraction, the percentage of radiation in the GI tract relative to the whole animal was calculated by dividing the counts in the GI tract by those in the whole animal and then multiplying by 100.

### Statistics

All values are expressed as mean ± standard deviation (SD). Tests for significance were performed in GraphPad Prism version 6.0c for Mac OS X. For comparisons between two groups, Student's t-test and the F test for equal sample variance were performed. For comparisons among multiple groups, one- way analysis of variance (ANOVA) with Tukey's post test and the Brown-Forsythe test for equal sample variance were performed. When the sample variances were determined to be unequal, Welch's correction was applied to t-tests and the Kruskal-Wallis test followed by the Dunn Multiple Comparison post test was performed on data analyzed by one-way ANOVA. Pearson's chi-squared test was used to compare actual versus expected genotype ratios obtained in our breedings. A P value of 0.05 percent or less was considered significant.

## Results

### Generation of hephaestin knockout mice

**Figure 1:**
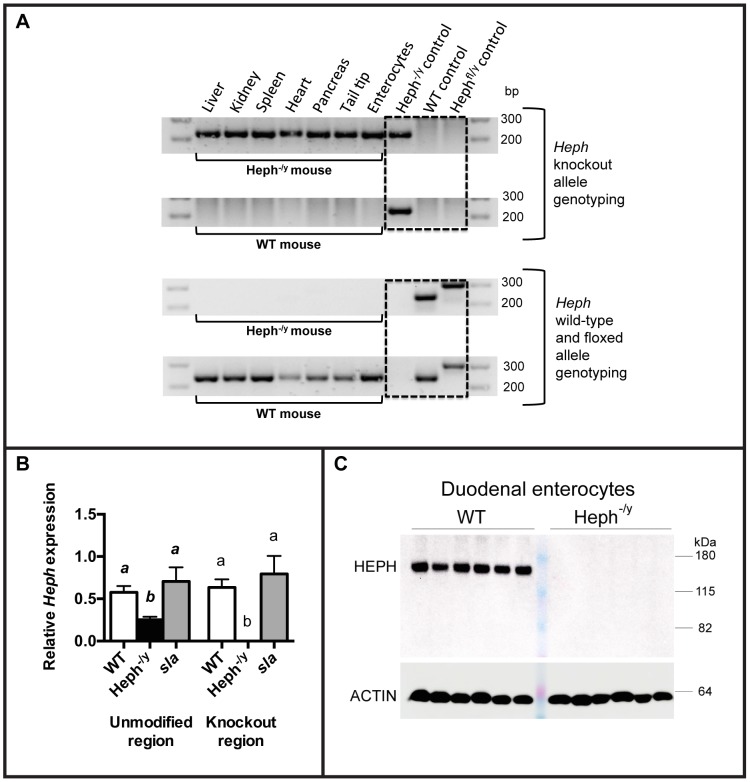
Verification of whole body hephaestin knockout. **A.** DNA extracted from a panel of homogenized tissues from Heph^-/y^ and WT littermates was amplified by PCR. Top panel, results for the “*Heph* knockout” reaction, which yields a strong band at 230 bp if a *Heph* knockout allele is present. Bottom panel, results from the “*Heph* wild-type” reaction, which yields a strong band at 240 bp if wild-type *Heph* DNA is present and a band at 300 bp if *Heph* floxed DNA is present. Tissues from two WT and four Heph^-/y^ mice were tested. **B.** *Heph* mRNA expression in proximal enterocytes was analyzed by RT-qPCR of cDNA from Heph^-/y^ and WT littermates, and *sla* mice, at 6-7 weeks of age (N = 7–12 mice per group). The mean ± SD for results from primers targeting an unmodified region downstream of the knockout site are shown at left, and the mean ± SD from primers targeting the knockout region (exon 4-exon 5 junction) are shown at right. *Heph* mRNA expression results were normalized to the expression of the *Hprt* housekeeping gene. For each primer set, groups that share at least one letter are not significantly different. **C.** HEPH protein expression in enterocytes from Heph^-/y^ and WT littermates (N = 6 per group) was examined by SDS-PAGE followed by immunoblotting with the HEPH D4 antibody. Actin expression in the same samples is shown below.

HEPH knockout mice were successfully generated using the Cre-lox system. Knockout was verified by PCR genotyping in DNA from the liver, kidney, spleen, heart, pancreas, tail tip, and isolated duodenal enterocytes of Heph^-/y^ and WT control littermates (Figure 1A). As expected, complete knockout of exon 4 of *Heph* was observed in all Heph^-/y^ tissues examined, and only WT alleles were present in WT control tissues.

*Heph* transcript levels were examined in duodenal enterocytes from 6–7 week old adult male Heph^-/y^ and WT littermates and age-matched *sla* mice (Figure 1B). The targeted exon in *Heph* was undetectable in the Heph^-/y^ enterocytes but downstream exons were still present in Heph^-/y^ enterocytes at levels approximately half that of WT controls. For both primer sets, results for the *sla* mice were not significantly different from WT.

Knockout of HEPH was further confirmed at the protein level by immunoblotting using two different antibodies that target different regions of the protein (Figure 1C; results from one antibody are shown). No HEPH protein was detected in enterocytes from Heph^-/y^ mice even when immunoblots were highly overexposed.

### Hephaestin knockout mice are viable yet iron deficient

**Figure 2:**
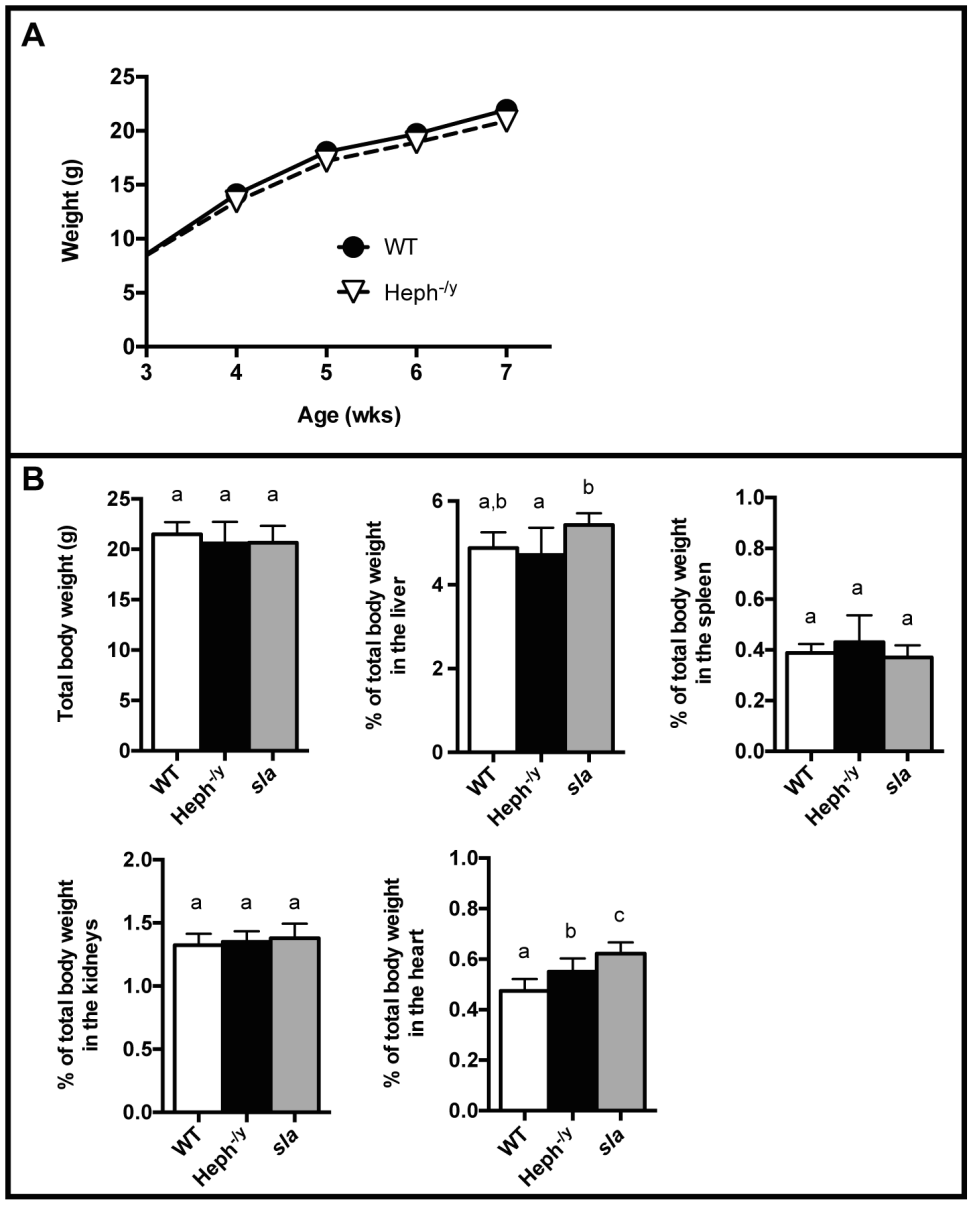
Body weight and relative tissue weights. **A**. Body weight time course for male Heph^-/y^ and WT littermates, N = 6–12 mice per age per genotype. **B**. Total body weight and the percentage of total body weight in various tissues in males at 6–7 weeks of age, N = 8–10 mice per group. **A**–**B.** Mean ± SD. Groups that share at least one letter are not significantly different.

HEPH whole body knockout mice were viable and appeared grossly normal at birth. Examination of the genotypes of 195 weaned pups (95 males, 100 females) from 34 litters born to 8 female heterozygotes (Heph^+/−^) bred with male Heph knockout hemizygotes, revealed slightly lower numbers of Heph^-/y^ versus WT and Heph^-/-^ versus heterozygous littermates (42% vs 58% for males and 47% vs 53% for females, respectively), but results were not statistically different from the expected 50∶50 ratios (P = 0.1238 and P = 0.5485 for males and females, respectively) as determined by Pearson's chi-squared test. Heph^-/y^ and WT male littermates exhibited no significant differences in body weight when weighed weekly from 3–7 weeks of age (Figure 2A). Heph^-/y^ and *sla* mice were not significantly different when compared to WT in total body weight or in the percentage of total body weight in the liver, spleen, or kidneys (Figure 2B) at 6–7 weeks of age. The heart, however, was slightly enlarged in both the Heph^-/y^ and *sla* mice when expressed as a percentage of total body weight.

**Figure 3:**
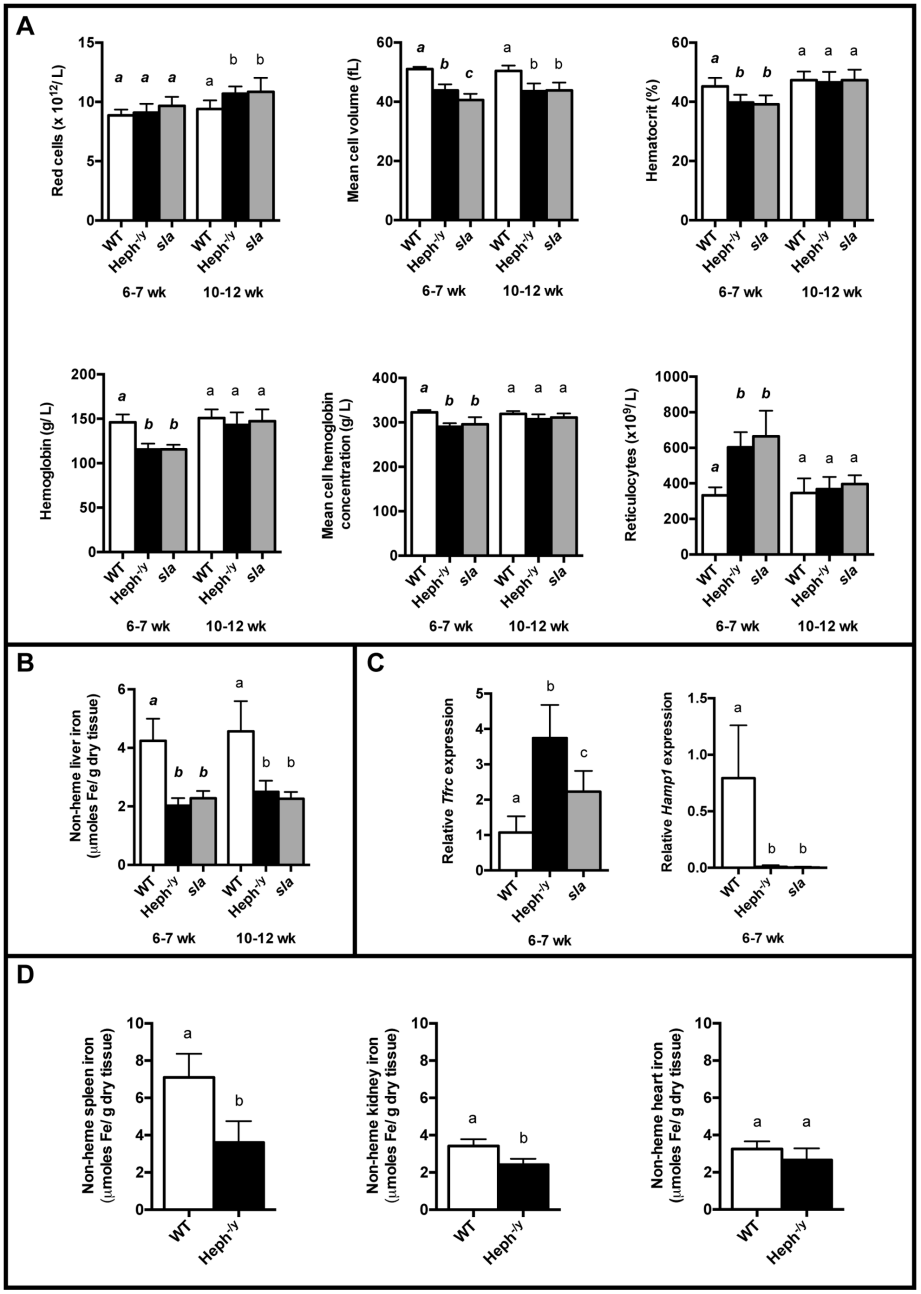
Hematology and tissue iron status. **A.** Hematology of 6–7 week old and 10–12 week old WT and Heph^-/y^ male littermates, and *sla* mice, N = 7–14 mice per group. **B.** Non-heme iron in livers from 6–7 week old and 10–12 week old Heph^-/y^ and WT littermates and *sla* mice, N = 7–17 mice per group. **C.** The expression of *Tfrc* and *Hamp1* mRNA as measured by RT-qPCR of cDNA from livers from 6–7 week old male Heph^-/y^ and WT littermates, and *sla* mice, N = 8–12 mice per group. The expression results were normalized to expression of the *Hprt* housekeeping gene. **D.** Non-heme iron in the spleen, kidney, and heart of young 6–7 week old male Heph^-/y^ and WT littermates, N = 7 mice per group. **A**–**D**. Mean ± SD. For each age range, groups that share at least one letter are not significantly different.

Adult Heph^-/y^ mice had a hypochromic, microcytic anemia, but the anemia resolved as the animals aged (Figure 3A). At 6–7 weeks of age, Heph^-/y^ and *sla* male mice had approximately the same number of red cells as WT controls, but the red cells of Heph^-/y^ and *sla* mice were significantly smaller and made up a smaller fraction of the total blood volume (lower hematocrit). Heph^-/y^ and *sla* mouse blood also contained significantly less hemoglobin overall, and the average concentration of hemoglobin in the red cells of these mice was low. Both genotypes also had nearly twice the number of reticulocytes as WT controls. Older (10–12 weeks of age) male Heph^-/y^ and *sla* mice were no longer anemic. Heph^-/y^ and *sla* mice had significantly more red cells than WT controls, but the red cells were still, on average, microcytic. The average concentration of hemoglobin per red cell no longer differed from that of WT mice and neither the hematocrit nor the total amount of hemoglobin in the blood was lower in 10–12 week old Heph^-/y^ and *sla* mice than in WT controls.

Liver non-heme iron levels were lower in Heph^-/y^ and *sla* mice relative to controls at both 6–7 and 10–12 weeks of age (Figure 3B). The expression of liver *Tfrc* mRNA, which inversely correlates with tissue iron levels, was significantly greater in 6–7 week old Heph^-/y^ mice than in WT littermates (Figure 3C). Liver *Tfrc* expression in *sla* mice was also significantly greater than in WT mice, but expression was not as high as in Heph^-/y^ mice. In addition, liver *Hamp1* mRNA expression was undetectable in both Heph^-/y^ and *sla* mice, consistent with both the low liver iron levels in these mice as well as their anemia. Iron levels in spleen and kidney were also tested in 6–7 week old Heph^-/y^ and WT littermates and were significantly lower in Heph^-/y^ mice (Figure 3D). Heart iron levels were not significantly different, however.

### Hephaestin knockout mice exhibit defects in intestinal iron absorption

**Figure 4:**
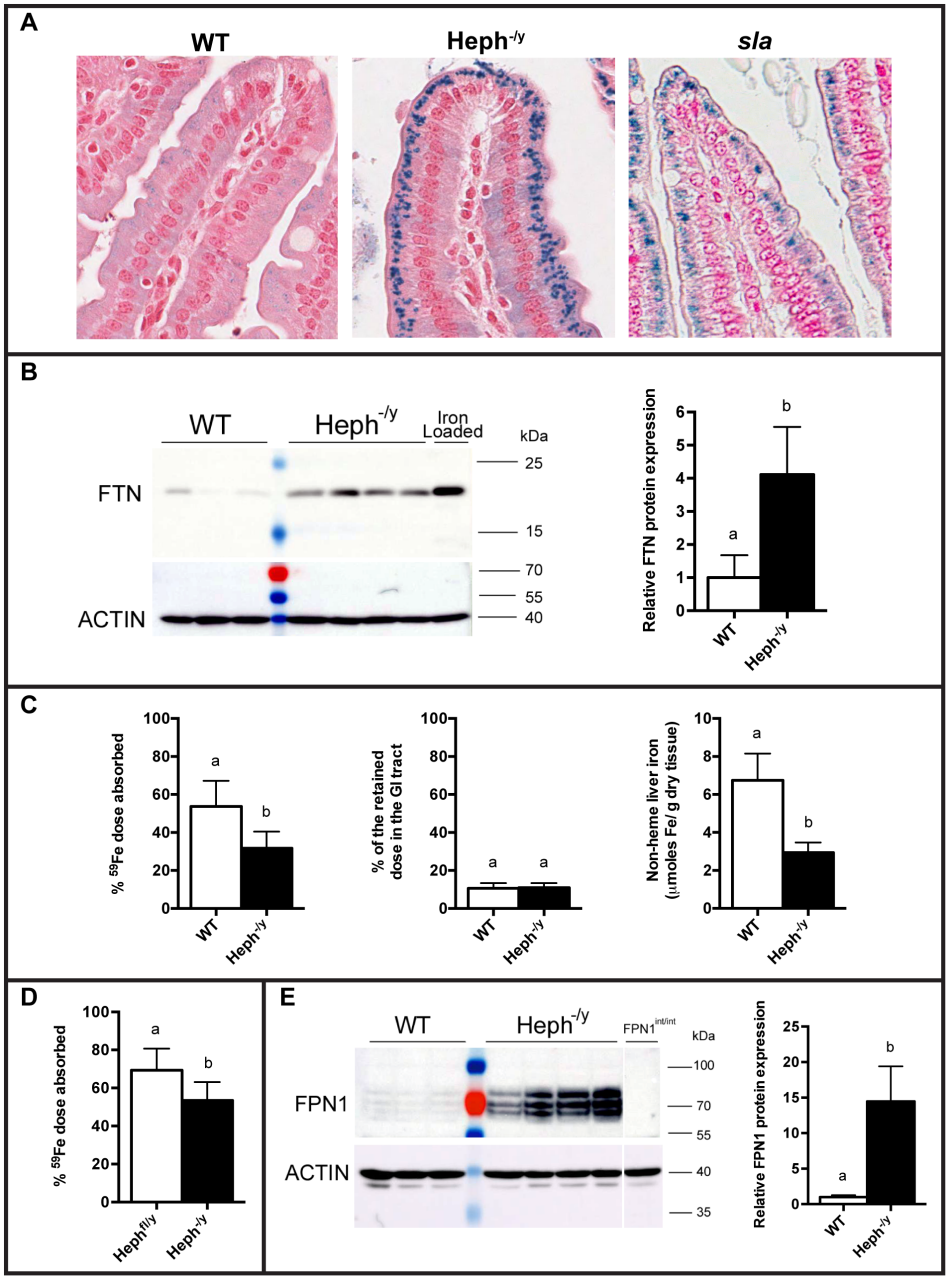
Intestinal iron retention and absorption. **A.** Representative Perls' Prussian blue stained duodenal sections from male mice maintained on a standard rodent chow diet. Aperio Scanscope XT, 40× magnification of a villus tip. Non-heme iron stains blue. **B.** FTN protein expression in duodenal enterocytes from WT and Heph^-/y^ male mice at 6–7 weeks of age, and from iron loaded control enterocytes from an intestine-specific FPN1 knockout mouse, was examined by SDS-PAGE followed by immunoblotting. Actin expression in the same samples is shown below. At right, densitometry data from this blot and another (N = 9–10 total mice per genotype). **C.** Male Heph^-/y^ (N = 5) and WT littermates (N = 5) 9–16 weeks old were put on an iron-deficient diet for one week and then dosed by gavage with ^59^Fe. Left, the percentage of the dose retained five days after dosing. Middle, the percentage of the total retained dose that was in the GI tract five days after dosing; as expected given the rapid turnover of enterocytes, levels were low and not significantly different between the genotypes. Right, total non-heme liver iron as measured by colorimetric assay. **D.** Whole body iron absorption in mice following dietary-induced stimulation of absorption. Weanling male Heph^-/y^ (N = 8) and Heph^fl/y^ (N = 7) controls (3 weeks old) were placed on an iron-deficient diet six weeks prior to dosing with ^59^Fe, and then were sacrificed five days after the dose. The dose retained in the GI tract was negligible and not different between the groups (data not shown). **E.** FPN1 protein expression in duodenal enterocytes from WT and Heph^-/y^ male mice at 6–8 weeks of age, and from control enterocytes from an intestine-specific FPN1 knockout mouse, was examined by SDS-PAGE followed by immunoblotting. Actin expression in the same samples is shown below. Densitometry data from this blot are shown at the right. **B**–**E.** Mean ± SD. Groups that share at least one letter are not significantly different.

Perls' Prussian blue staining for ferric iron in the duodenum revealed strong iron deposits in the supranuclear region of enterocytes in Heph^-/y^ and *sla* mice, but not in wild-type animals (Figure 4A). Enterocytes in the upper third of the villi, which are the most highly differentiated population of enterocytes and contribute the most to iron absorption, showed strong accumulation of iron in the Heph^-/y^ and *sla* mice. Ferritin (FTN) protein, a marker of iron stores, was examined by immunoblotting and levels were approximately four times higher in Heph^-/y^ enterocytes than WT controls (Figure 4B).

Iron absorption was determined by whole body counting five days after gavage with a dose of radioactive iron (Figure 4C). When adult male Heph^-/y^ and WT littermates were put on an iron-deficient diet for one week prior to dosing, Heph^-/y^ mice absorbed significantly less iron than WT mice despite having liver non-heme iron stores less than half that of the WT animals. Very little radiolabeled iron was detected in the GI tract (stomach to anus) of any mice, and there were no differences between the levels of radioactive iron in the GI tract of WT and Heph^-/y^ mice (Figure 4C), consistent with turnover of the intestinal enterocytes, as expected, in the five day post-dose period. Strong stimulation of iron absorption induced by six weeks on an iron-deficient diet significantly upregulated iron absorption in Heph^-/y^ mice, indicating that adult mice lacking HEPH are able to increase iron absorption when body demands are very high (Figure 4D). Heph^-/y^ males, however, still had a significantly lower level of iron absorption than age matched controls. Interestingly, higher levels of the iron export protein FPN1 were found in duodenal enterocytes from Heph^-/y^ than WT controls at 6–8 weeks of age (Figure 4E).

### Intestine-specific hephaestin knockout mice have a similar, yet milder phenotype than whole body knockout mice

Similar to the Heph^-/y^ mice, Heph^int/y^ mice were viable and grossly indistinguishable from their Heph^fl/y^ littermates. By PCR genotyping of a panel of tissues, knockout of *Heph* was detected only in the enterocytes of Heph^int/y^ mice, and only floxed alleles were present in the other tissues (Figure S1). Floxed DNA was, however, also detected in the Heph^int/y^ enterocytes. This may be explained by either contamination from other intestinal cell types or by incomplete recombination, as has been reported previously in other intestinal knockout mice generated with this particular Cre transgene [31]. By immunoblot, most Heph^int/y^ mice had little to no detectable HEPH protein expression in isolated enterocytes (Figure S2). Heph^int/y^ mice that exhibited some HEPH expression in our enterocyte preparations, as determined by immunoblotting, were excluded from our analyses.

**Figure 5:**
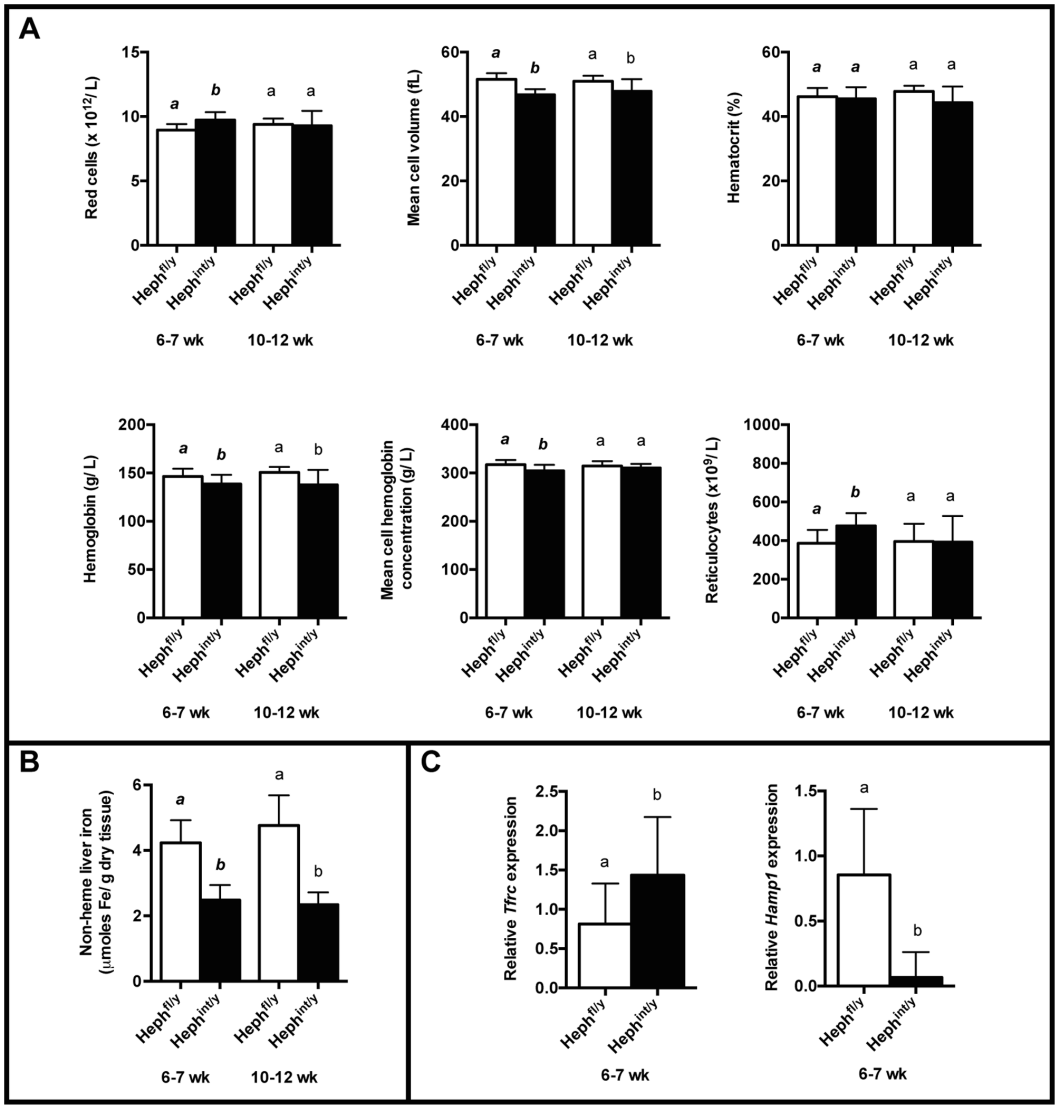
Hematology and iron status of mice with intestine-specific knockout of HEPH. **A.** Hematology of 6–7 week old and 10–12 week old Heph^int/y^ and Heph^fl/y^ male littermates. N = 8–19 mice per group. **B.** Non-heme iron in livers from 6–7 week old and 10–12 week old Heph^fl/y^ and Heph^int/y^ littermates, N = 9–23 mice per group. **C.** The expression of *Tfrc* and *Hamp1* mRNA was measured by RT-qPCR of cDNA from livers from 6–7 week old male Heph^fl/y^ and Heph^int/y^ littermates, N = 11–22 mice per genotype. The expression results were normalized to expression of the *Hprt* housekeeping gene. **A**–**C**. Mean ± SD. For each age group, groups that share at least one letter are not significantly different.

Mice with intestinal knockout of HEPH had a microcytic anemia at 6–7 weeks of age, and improvement in most hematological parameters was seen by 10–12 weeks of age (Figure 5A). Overall, however, their anemia was less severe than that of the Heph^-/y^ and *sla* mice. When hematology parameters were examined across all five groups (Heph^-/y^, *sla*, WT, Heph^int/y^, and Heph^fl/y^) at 6–7 weeks of age by one-way ANOVA followed by Tukey's post test, or the Kruskal-Wallis test followed by the Dunn Multiple Comparison post test where appropriate, Heph^-/y^ and *sla* mice had significantly smaller red cells, lower hemoglobin and hematocrit levels, and more reticulocytes than Heph^int/y^ mice, indicating a more severe anemia (Table 3). When a similar analysis was carried out across all five groups at 10–12 weeks of age, Heph^-/y^ and *sla* mice still had significantly smaller red cells than the Heph^int/y^, WT, and Heph^fl/y^ mice, suggesting that the anemia in the Heph^int/y^ mice had resolved more completely than in those animals where HEPH was disrupted globally. With the exception of *sla* mice having more red cells than Heph^int/y^ mice at 10–12 weeks of age, no other hematological parameters differed significantly between *sla*, Heph^-/y^, and Heph^int/y^ mice (Table 3).

**Table 3:** Hematology with comparisons across all genotypes.

Analysis	Age (wks)	Genotype				
		WT	Heph^-/y^	*sla*	Heph^fl/y^	Heph^int/y^
**Red blood cells (x 10^12^/L)**	6–7	8.9±0.5^a,b^ (N = 8)	9.1±0.7^a,b,c^ (N = 10)	9.7±0.8^a,c^ (N = 10)	9.0±0.5^b^ (N = 18)	9.7±0.6^c^ (N = 8)
	10–12	9.4±0.7^a^ (N = 7)	10.7±0.6^b,c^ (N = 9)	10.9±1.2^b^ (N = 14)	9.4±0.5^a^ (N = 15)	9.3±1.1^a,c^ (N = 9)
**Mean cell volume (fL)**	6–7	51±1^a^ (N = 8)	44±2^b^ (N = 10)	41±2^c^ (N = 10)	52±2^a^ (N = 18)	47±2^d^ (N = 8)
	10–12	50±2^a,b^ (N = 7)	44±3^c^ (N = 9)	44±3^c^ (N = 14)	51±2^a^ (N = 15)	48±4^b^ (N = 9)
**Hematocrit (%)**	6–7	45±3^a^ (N = 8)	40±3^b^ (N = 10)	39±3^b^ (N = 10)	46±3^a^ (N = 18)	46±4^a^ (N = 8)
	10–12	47±3^a^ (N = 7)	47±4^a^ (N = 9)	47±4^a^ (N = 14)	48±2^a^ (N = 15)	44±5^a^ (N = 9)
**Hemoglobin (g/L)**	6–7	146±9^a^ (N = 8)	115±7^b^ (N = 10)	116±5^b^ (N = 10)	146±8^a^ (N = 18)	139±10^a^ (N = 8)
	10–12	151±10^a^ (N = 7)	143±14^a^ (N = 9)	147±13^a^ (N = 14)	151±6^a^ (N = 15)	138±16^a^ (N = 9)
**Mean cell hemoglobin (pg)**	6–7	16.5±0.2^a^ (N = 8)	12.7±0.7^b^ (N = 10)	12.0±0.9^b^ (N = 10)	16.3±0.4^a^ (N = 18)	14.2±0.7^a,b^ (N = 8)
	10–12	16.1±0.4^a^ (N = 7)	13.4±1.1^b^ (N = 9)	13.6±0.6^b^ (N = 14)	16.0±0.5^a^ (N = 15)	14.9±1.2^a,b^ (N = 9)
**Mean cell hemoglobin concentration (g/L)**	6–7	323±5^a^ (N = 8)	290±8^b^ (N = 10)	296±16^b^ (N = 10)	317±10^a^ (N = 18)	305±12^a,b^ (N = 8)
	10–12	319±6^a^ (N = 7)	307±11^a^ (N = 9)	311±9^a^ (N = 14)	315±10^a^ (N = 15)	311±8^a^ (N = 9)
**Reticulocytes (x 10^9^/L)**	6–7	333±45^a^ (N = 7)	603±85^b^ (N = 7)	665±144^b^ (N = 9)	386±69^a,c^ (N = 16)	476±66^c^ (N = 10)
	10–12	345±83^a^ (N = 7)	368±68^a^ (N = 8)	397±48^a^ (N = 8)	394±92^a^ (N = 19)	392±136^a^ (N = 11)

Mean ± SD. N =  number of mice analyzed. Results from WT, Heph^-/y^, *sla*, Heph^fl/y^, and Heph^int/y^ mice were analyzed together by one-way ANOVA and Tukey's post test, or when appropriate, by the Kruskal-Wallis test followed by the Dunn Multiple Comparison post test. (Note that, in contrast to the statistical analysis results shown here, the statistical analysis results shown in Figures 3A and 5A are only for comparisons between the genotypes in those figures.) Separate statistical analyses were done for each age group. For each analysis, groups that share at least one superscripted letter are not significantly different.

**Figure 6:**
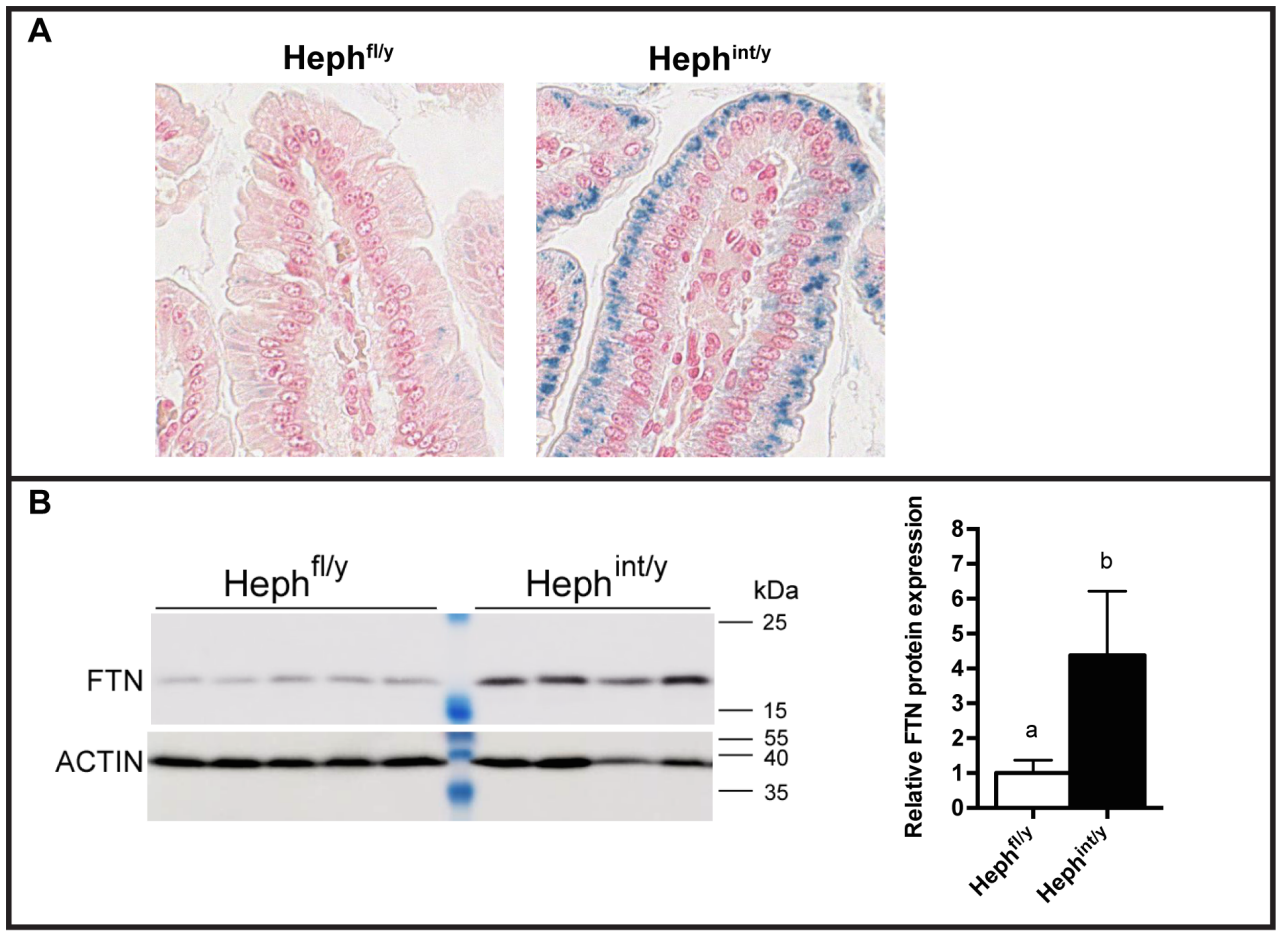
Intestinal iron loading in mice with intestine-specific knockout of HEPH. **A.** Representative Perls' Prussian blue stained duodenal sections from male mice maintained on a chow diet. Aperio Scanscope XT, 40× magnification of a villus tip. Non-heme iron stains blue. **B.** FTN protein expression in duodenal enterocytes from Heph^fl/y^ and Heph^int/y^ male mice at 6–7 weeks of age as examined by SDS-PAGE followed by immunoblotting. Actin expression in the same samples is shown below. At right, densitometry data is shown for this blot. Mean ± SD. Groups that share at least one letter are not significantly different.

Liver iron stores in Heph^int/y^ mice, as for the Heph^-/y^ and *sla* mice, were about half that of controls at 6–7 weeks of age and remained low at 10–12 weeks of age (Figure 5B). Liver *Tfrc* mRNA levels at 6–7 weeks of age were approximately two-fold greater than in Heph^fl/y^ controls, and liver *Hamp1* mRNA expression was detectable, but was significantly lower than in Heph^fl/y^ controls (Figure 5C). Iron loading and increased FTN expression were seen in duodenal enterocytes by Perls' staining and immunoblotting, respectively (Figures 6A and 6B). The expression of divalent metal transporter 1 (DMT1), the apical iron import protein, was also measured at the mRNA level in 6–7 week old male mice using a set of primers that amplified all four major isoforms. Results (Figure S3) were consistent with those reported by us previously in *sla* mice [25]. *Dmt1* expression levels in isolated proximal small intestinal enterocytes of anemic Heph^-/y^, *sla*, and Heph^int/y^ mice were similar to those of non-anemic WT and Heph^fl/y^ controls, likely due to the regulation of DMT1 by local iron levels in the enterocyte rather than systemic iron status, as noted in the study by Chen et al. [25].

## Discussion

The mechanisms by which dietary iron is absorbed and transferred from intestinal enterocytes to the blood are complex, and details are still emerging as to the identity and roles of proteins involved in this process. Our results demonstrate that, while the MCF HEPH is important for optimal iron absorption, it is not essential.

Young adult Heph^-/y^ mice exhibited a hypochromic, microcytic anemia that improved with age, characteristics similar to those reported previously for *sla* mice [18]. At 6–7 weeks of age, Heph^-/y^ mice were significantly iron-deficient, as indicated by reduced liver *Hamp1* expression, low tissue iron, and elevated *Tfrc* expression in the liver. The hearts of Heph^-/y^ mice were also enlarged relative to WT littermates, likely as a response to their anemia [32]. The phenotypes of age-matched Heph^-/y^ and *sla* mice were generally indistinguishable in the studies performed here, suggesting that the *sla* deletion abolishes HEPH function despite, as shown previously, preserving some of the protein's *in vitro* oxidase activity [19]. This loss of function might be explained by the mislocalization of the mutated HEPH protein in *sla* mice [33]. Heph^int/y^ mice had a slightly milder phenotype than Heph^-/y^ mice. The iron status of Heph^-/y^, Heph^int/y^, and *sla* mice improved as the mice aged, but liver non-heme iron levels still remained lower in these mice than in controls. The more severe iron deficiency phenotype observed in young relative to older knockout and mutant mice is likely explained by the greater iron requirements of younger animals due to their rapid growth.

The iron deficiency phenotype of the Heph^-/y^ and Heph^int/y^ mice, coupled with the dramatic iron loading that was observed in the supranuclear region of Heph^-/y^ and Heph^int/y^ enterocytes by Perls' stain, suggests defects in iron absorption, as has been reported in *sla* mice [34]. We performed several iron absorption studies to confirm this finding. After being challenged with an iron-deficient diet for one week, adult Heph^-/y^ mice absorbed significantly less radioactive iron than controls. When mice were challenged with an iron-deficient diet for six weeks from weaning, both groups absorbed high amounts of the iron dose, although Heph^-/y^ mice still absorbed less than controls. These findings suggest that Heph^-/y^ mice are able to upregulate their iron absorption, but not as effectively as WT mice.

Studies in other cell types have indicated that the iron export protein FPN1 can be internalized and degraded in the absence of ferroxidase activity, leading to cellular iron loading and decreased iron export [2], [4]. While this could be occurring to some extent in Heph^-/y^ enterocytes, levels of FPN1 were significantly greater in duodenal enterocytes from Heph^-/y^ mice than WT controls. Similar results were also reported in *sla* mice [18]. The low levels of hepcidin expression coupled with the high intracellular iron levels in the enterocytes of Heph^-/y^ and *sla* mice likely leads to both increased FPN1 expression and decreased hepcidin-mediated FPN1 turnover. The increased enterocyte FPN1 protein levels, however, are still not sufficient to maintain normal iron absorption in Heph^-/y^ mice. Further studies are thus needed to determine precisely how lack of HEPH leads to decreased intestinal iron absorption.

Additional ferroxidases may compensate for the lack of HEPH in the intestine. A cytosolic ferroxidase activity has been detected in extracts from isolated enterocytes from Heph^-/y^ mice, but the source and role of that activity in intestinal iron absorption remains uncertain [35]. Circulating CP may be able to partially compensate for lack of HEPH, as CP facilitates the release of iron from other tissues and has been previously shown in mice to augment intestinal iron absorption after severe blood loss [8]. However, we were not able to detect any differences by immunoblot in the amount of CP in the serum of Heph^-/y^ versus WT controls (data not shown), suggesting that systemic upregulation of CP is not involved. Future studies in mice with double knockout of CP and HEPH will allow us to better address the role of CP in intestinal iron absorption. More recently, a third MCF, zyklopen, was identified which is expressed in some tissues but not intestinal enterocytes, so it is unlikely to compensate for loss of HEPH [36].

The similar phenotypes of the Heph^-/y^ and Heph^int/y^ mice indicate that HEPH function in intestinal enterocytes alone is particularly important for maintaining systemic iron homeostasis. This observation is consistent with the critical role of these cells in body iron acquisition, as well as the high level of HEPH expression in this tissue. However, the milder presentation of Heph^int/y^ mice, as revealed in their hematology and liver *Hamp1* expression, indicates that HEPH plays additional extra-intestinal roles in iron metabolism.

If HEPH is involved in efficient iron efflux from other cell types, it might be expected that some other cell types would show iron accumulation in the whole body knockout model. Currently HEPH is only known to play an important role in the intestine, eye, and brain, with cells in these tissues accumulating iron when HEPH expression is perturbed [12], [22]. With the exception of duodenal enterocytes, however, no dramatic increases in iron loading in any cell types were observed by Perls' staining in age-matched panels of tissues (liver, heart, spleen, pancreas, kidney, ileum, jejunum, colon, lung, and testes) from Heph^-/y^ versus control mice at 10–12, 23–26 or 76–79 weeks of age (data not shown). The brain and eyes, however, were not studied. Because HEPH is also ablated in the intestine of Heph^-/y^ mice, less iron is available to load other cells in the body, and thus any iron-loading phenotypes may not be readily apparent without administration of parenteral iron to bypass intestinal iron absorption. As has been shown in the eye, some cell types express other ferroxidases that could at least partially compensate for the lack of HEPH, so double knockout models may provide a more sensitive means to determine the importance of HEPH in other cell types [22].

## Supporting Information

Figure S1 **Verification of intestine-specific knockout of** ***Heph*** **at the DNA level.** DNA extracted from a panel of homogenized tissues from Heph^fl/y^ and Heph^int/y^ littermates was amplified by PCR. Top panel, results for the “*Heph* knockout” reaction, which yields a strong band at 230 bp if a Heph knockout allele is present. Bottom panel, results from the “*Heph* wild-type” reaction, which yields a strong band at 240 bp if a wild-type *Heph* DNA is present and a band at 300 bp if *Heph* floxed DNA is present. Tissues from two mice of each genotype were tested.
(TIF)

Figure S2 **Verification of HEPH knockout in the enterocytes of Heph^int/y^ mice.** HEPH protein expression in enterocytes from Heph^fl/y^ and Heph^int/y^ mice (N = 6 per genotype) was examined by SDS-PAGE followed by immunoblotting with the HEPH D4 antibody. Actin expression in the same samples is shown below.
(TIF)

Figure S3 ***Dmt1*** **mRNA levels in the enterocytes of Heph^-/y^,** ***sla*** **, and Heph^int/y^ mice, and Heph^fl/y^ and WT controls.** The expression of *Dmt1* mRNA as measured by RT-qPCR using cDNA prepared from RNA from isolated proximal small intestinal enterocytes from 6–7 week old male mice, N = 11–19 mice per genotype. *Dmt1* expression was normalized to the expression of the *Hprt* housekeeping gene. Results are presented as mean ± SD. Statistical analysis was carried out across all five groups by the Kruskal-Wallis test followed by the Dunn Multiple Comparison post test. Groups that share at least one letter are not significantly different.
(TIF)
