# Supplementary figures and images for: The Multicopper Ferroxidase Hephaestin Enhances Intestinal Iron Absorption in Mice
Source: PLoS One. 2014 Jun 4;9(6):e98792. doi: 10.1371/journal.pone.0098792 (PMC4045767; doi:10.1371/journal.pone.0098792)

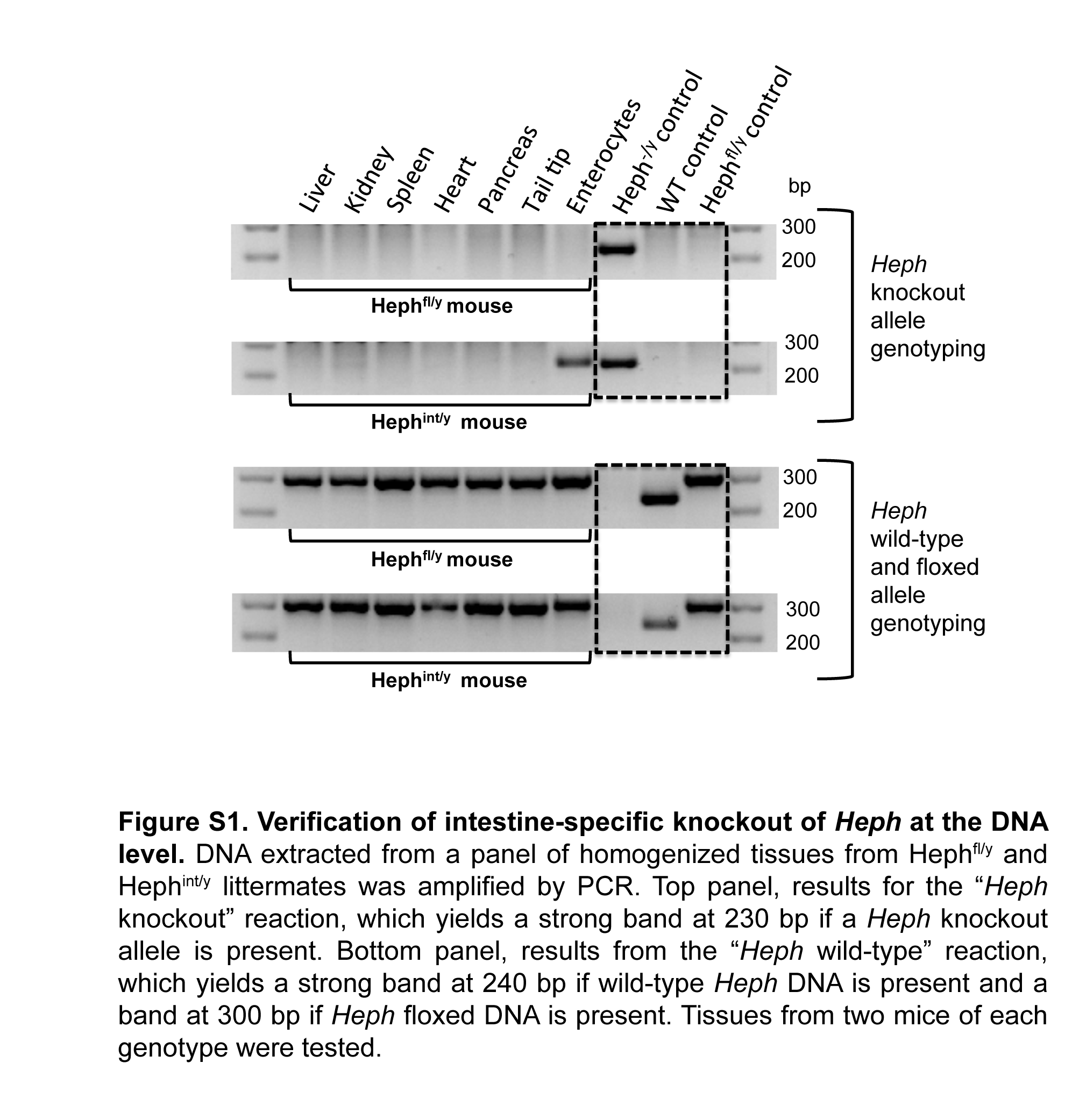

Supplement: Figure S1 — Verification of intestine-specific knockout of Heph at the DNA level. DNA extracted from a panel of homogenized tissues from Hephfl/y and Hephint/y littermates was amplified by PCR. Top panel, results for the “Heph knockout” reaction, which yields a strong band at 230 bp if a Heph knockout allele is present. Bottom panel, results from the “Heph wild-type” reaction, which yields a strong band at 240 bp if a wild-type Heph DNA is present and a band at 300 bp if Heph floxed DNA is present. Tissues from two mice of each genotype were tested. (TIF) [file pone.0098792.s001.tif]

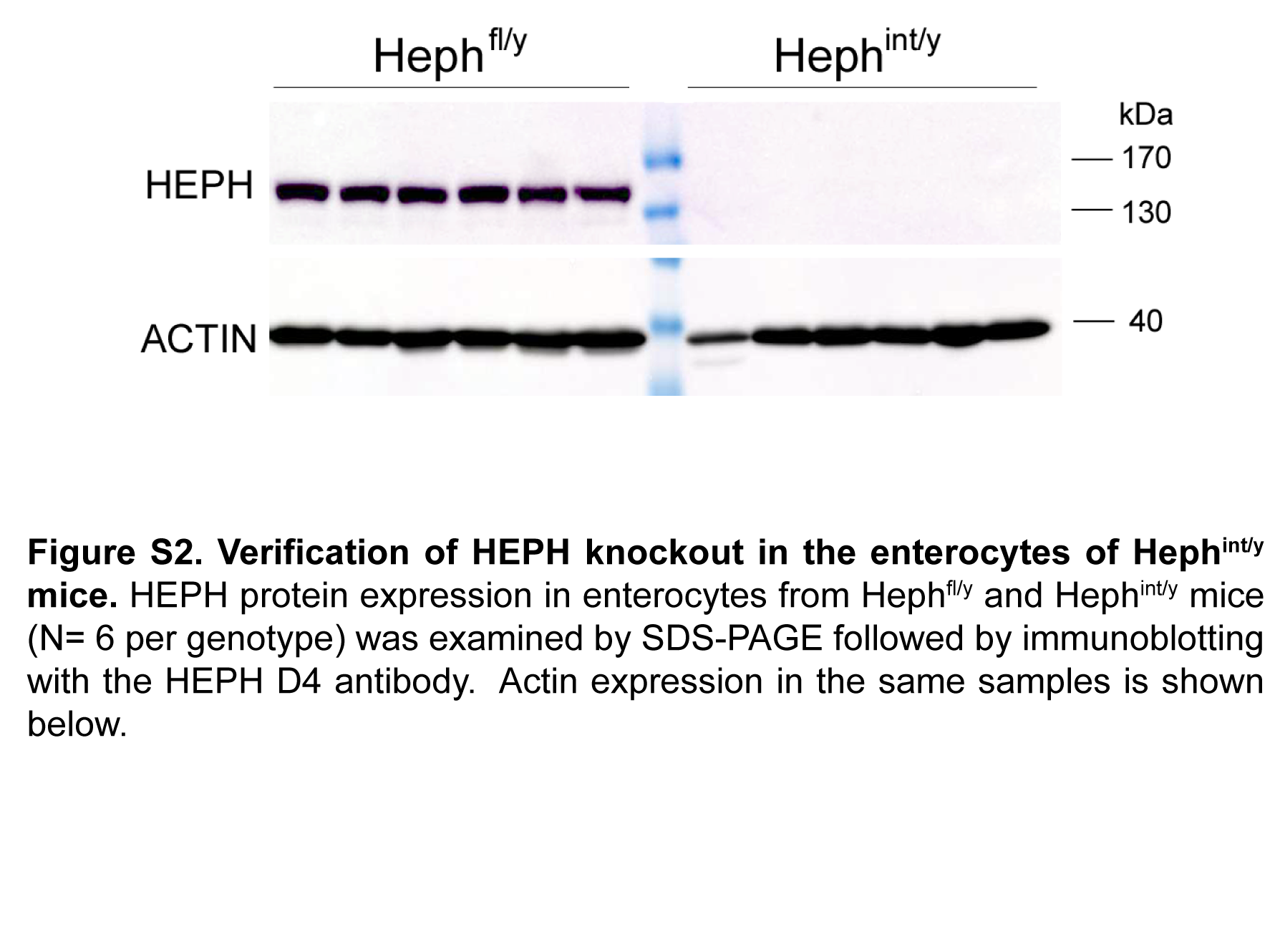

Supplement: Figure S2 — Verification of HEPH knockout in the enterocytes of Hephint/y mice. HEPH protein expression in enterocytes from Hephfl/y and Hephint/y mice (N = 6 per genotype) was examined by SDS-PAGE followed by immunoblotting with the HEPH D4 antibody. Actin expression in the same samples is shown below. (TIF) [file pone.0098792.s002.tif]

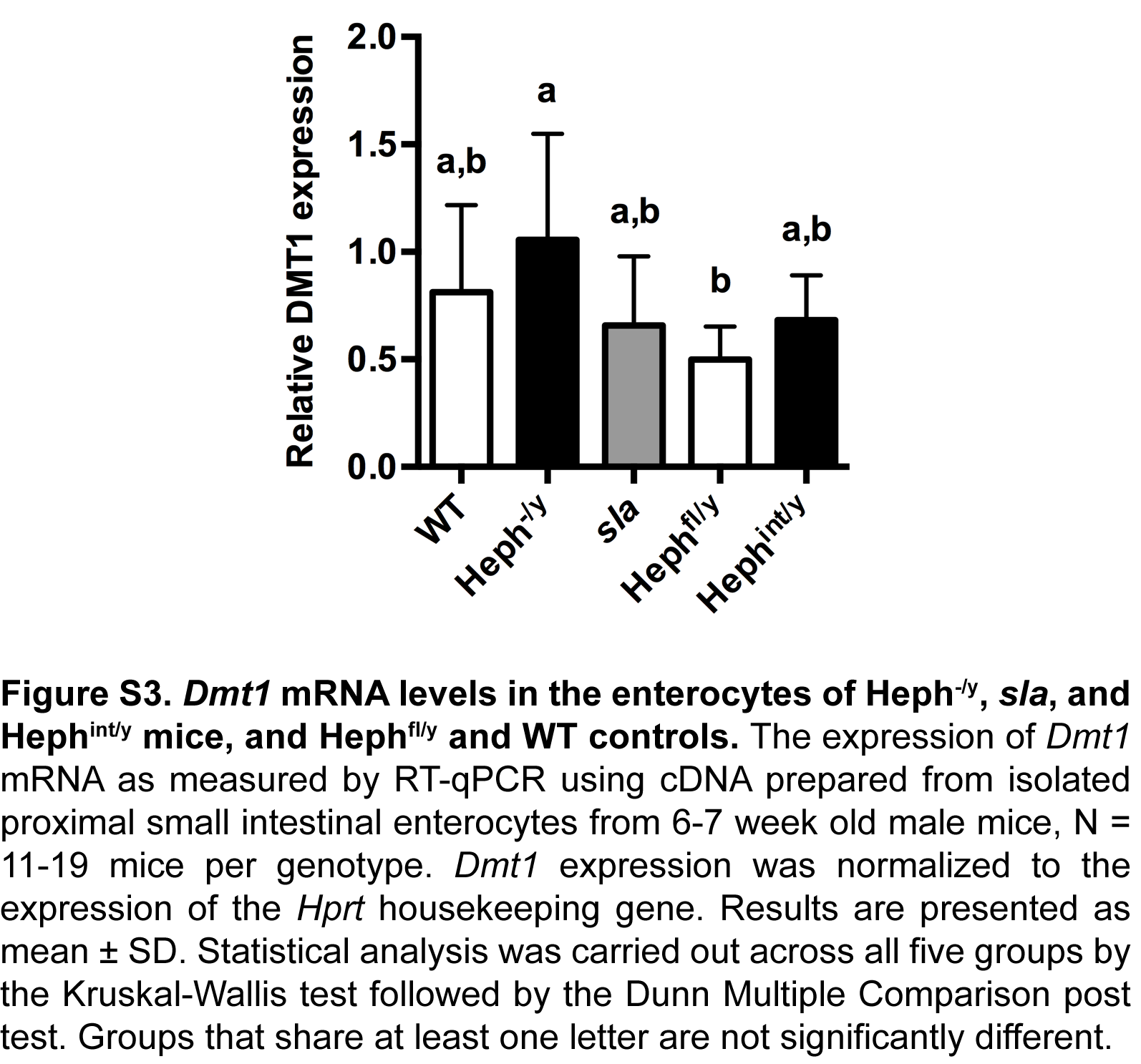

Supplement: Figure S3 — Dmt1 mRNA levels in the enterocytes of Heph-/y, sla , and Hephint/y mice, and Hephfl/y and WT controls. The expression of Dmt1 mRNA as measured by RT-qPCR using cDNA prepared from RNA from isolated proximal small intestinal enterocytes from 6–7 week old male mice, N = 11–19 mice per genotype. Dmt1 expression was normalized to the expression of the Hprt housekeeping gene. Results are presented as mean ± SD. Statistical analysis was carried out across all five groups by the Kruskal-Wallis test followed by the Dunn Multiple Comparison post test. Groups that share at least one letter are not significantly different. (TIF) [file pone.0098792.s003.tif]
